# Cross-talk between p21-activated kinase 4 and ERα signaling triggers endometrial cancer cell proliferation

**DOI:** 10.18632/oncotarget.19188

**Published:** 2017-07-12

**Authors:** Tao Su, Jun-Jie Qu, Kai Wang, Bi-Lan Li, Dong Zhao, Yi-Ping Zhu, Lei Ye, Wen Lu, Xiao-Ping Wan

**Affiliations:** ^1^ Department of Gynecology, Shanghai First Maternity and Infant Hospital, Tongji University School of Medicine, Shanghai, P.R. China; ^2^ Department of Gynecology, The International Peace Maternity & Child Health Hospital, Shanghai Jiao Tong University School of Medicine, Shanghai, P.R. China; ^3^ Clinical and Translational Research Center, Shanghai First Maternity and Infant Hospital, Tongji University School of Medicine, Shanghai, P.R. China

**Keywords:** endometrial carcinoma, p21-activated kinase 4 (Pak4), estrogen receptor alpha (ERα), cross-talk, proliferation

## Abstract

Cross-talk between estrogen receptor alpha (ERα) and signal transduction pathways plays an important role in the progression of endometrial cancer (EC). Here, we show that 17β-estradiol (E_2_) stimulation increases p21-activated kinase 4 (Pak4) expression and activation in ER-positive EC cells. The estrogen-induced Pak4 activation is mediated via the PI3K/AKT pathway. Estrogen increases Pak4 and phosphorylated-Pak4 (p-Pak4) nuclear accumulation, and Pak4 in turn enhances ERα trans-activation. Depletion or functional inhibition of Pak4 abrogates EC cell proliferation induced by E_2_, whereas overexpression of Pak4 rescues cell proliferation decreased by inhibiting the estrogen pathway. Pak4 knockdown decreases cyclin D1 expression and induces G1-S arrest. Importantly, Pak4 suppression inhibits E_2_ induced EC tumor growth *in vivo*, in a mouse xenograft model. These data demonstrate that estrogen stimulation increases Pak4 expression and activation, which in turn enhances ERα transcriptional activity and ERα-dependent gene expression, resulting in increased proliferation of EC cells. Thus inhibition of Pak4-ERα signaling may represent a novel therapeutic strategy against endometrial carcinoma.

## INTRODUCTION

Endometrial carcinoma (EC) is the most common malignancy of the female genital tract [1–3]. Estrogen stimulation is an important pathogenic factor contributing to endometrial carcinoma [4, 5]. Estrogen receptors (ERα and ERβ) are ligand-dependent transcription factors that mediate the effects of estrogen via gene regulation [6]. In addition, ERα can initiate several non-genomic signaling events in an estrogen-independent manner via post-translational modifications, such as phosphorylation. The cross-talk between ERα and signal transduction pathways leads to a series of cellular effects, including adhesion, migration, survival, and proliferation, which play a key role in tumorigenesis [7, 8].

The p21-activated kinases (Paks) are a family of serine/threonine kinases comprising six isoforms (group I includingPak1-3, and group II including Pak4-6) in humans, based on their structure and function [9, 10]. Interestingly, Paks have been found to mediate tamoxifen resistance in breast cancer [11, 12]. Pak1 signaling promotes trans-activation of ERα in breast cancer cells by phosphorylating ERα at serine 305 in the absence of its ligand estrogen, resulting in tamoxifen resistance [11]. Pak4 binds to ERα and promotes its transcriptional activity by phosphorylating ERα-Ser 305, and ERα in turn binds to the *Pak4* promoter and induces Pak4 transcription. The Pak4-ERα interaction decreases sensitivity to tamoxifen in MCF-7 human breast cancer cells [12]. However, tamoxifen use increases the risk of endometrial cancer due to its estrogenic effects on the endometrium [13, 14], suggesting different regulatory mechanisms of estrogen signaling in breast and endometrial cancer.

Pak4 is one of the major downstream kinases in oncogenic signaling [15, 16]. Pak4 is upregulated and activated by various stimuli [17, 18]. For example, it promotes prostate cancer cell migration in response to hepatocyte growth factor (HGF) [19]. In gestational trophoblastic disease, Pak4 is activated by human chorionic gonadotropin (hCG) via PI3K/PKB signaling [20]. We have previously demonstrated that the Pak4 expression increases with the progression of EC [21]. Furthermore, we have observed a nuclear localization of Pak4, especially the activated, phosphorylated Pak4 form (p-Pak4ser^474^) in endometrial cancer tissues [21], suggesting that Pak4 might activate ERα and contribute to estrogen-induced EC pathogenesis.

To explore this possibility, we have investigated the relationship between Pak4 and estrogen signaling in endometrial cancer. We tested the hypothesis that a positive feedback loop exists in which estrogen stimulates Pak4 expression and activation, which in turn promotes ERα trans-activation, and endometrial cancer cell proliferation. This feedback loop also involves PI3K/AKT signaling, cyclin D1, and cell cycle progression. These studies define a novel mechanism underlying estrogen signaling regulation, and suggest that Pak4 might be an important therapeutic target in endometrial cancer.

## RESULTS

### Estrogen up-regulates Pak4 expression and activation

**Figure 1:**
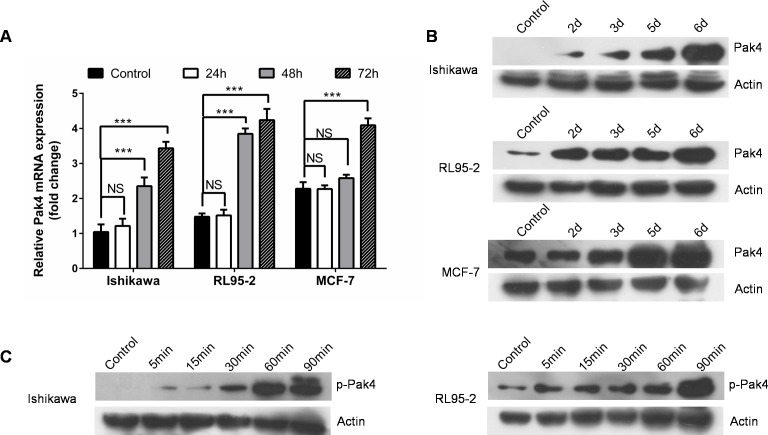
Estrogen increases Pak4 expression and activation **(A-B)** E_2_ induces Pak4 mRNA and protein levels. Serum-starved Ishikawa, RL95-2 and MCF-7 cells were treated with 10 nM E_2_, and cells were harvested at the indicated time. **(A)** The levels of Pak4 mRNA were determined by qRT-PCR, using β-actin as an internal control. Values represent mean ± s.d. (n = 3). ***P<0.001 compared with control. **(B)** The protein levels of Pak4 were assessed by Western blot. **(C)** Ishikawa and RL95-2 cells were serum-starved for 24 h, and then treated with 10 nM E_2_ for indicated times. The levels of p-Pak4ser^474^ were measured by Western blot, using β-actin as a loading control. All experiments were carried out in triplicates.

ER-positive human Ishikawa and RL95-2 endometrial cancer cells, as well as estrogen-responsive breast cancer MCF-7 cells were treated with a low-dose E_2_ (10 nM). We observed that E_2_ treatment led to a time-dependent increase in both Pak4 mRNA and protein levels (Figure 1A and 1B). In Ishikawa cells, the Pak4 protein levels started to rise after 2 days, and gradually peaked in 6 days. Similar trends were also found in RL95-2 cells. As for MCF-7 breast cancer cells, the levels of Pak4 mRNA and protein increased after 3 days of E_2_ stimulation.

Western blotting revealed a time-dependent increase in the levels of p-Pak4 Ser^474^ (the activated form) in Ishikawa and RL95-2 cells in the presence of E_2_. The level of p-Pak4 was increased after 5 min of E_2_ stimulation, and lasted for at least 90 min (Figure 1C), indicating that estrogen activates Pak4.

### Estrogen activates Pak4 via PI3K/AKT signaling

**Figure 2:**
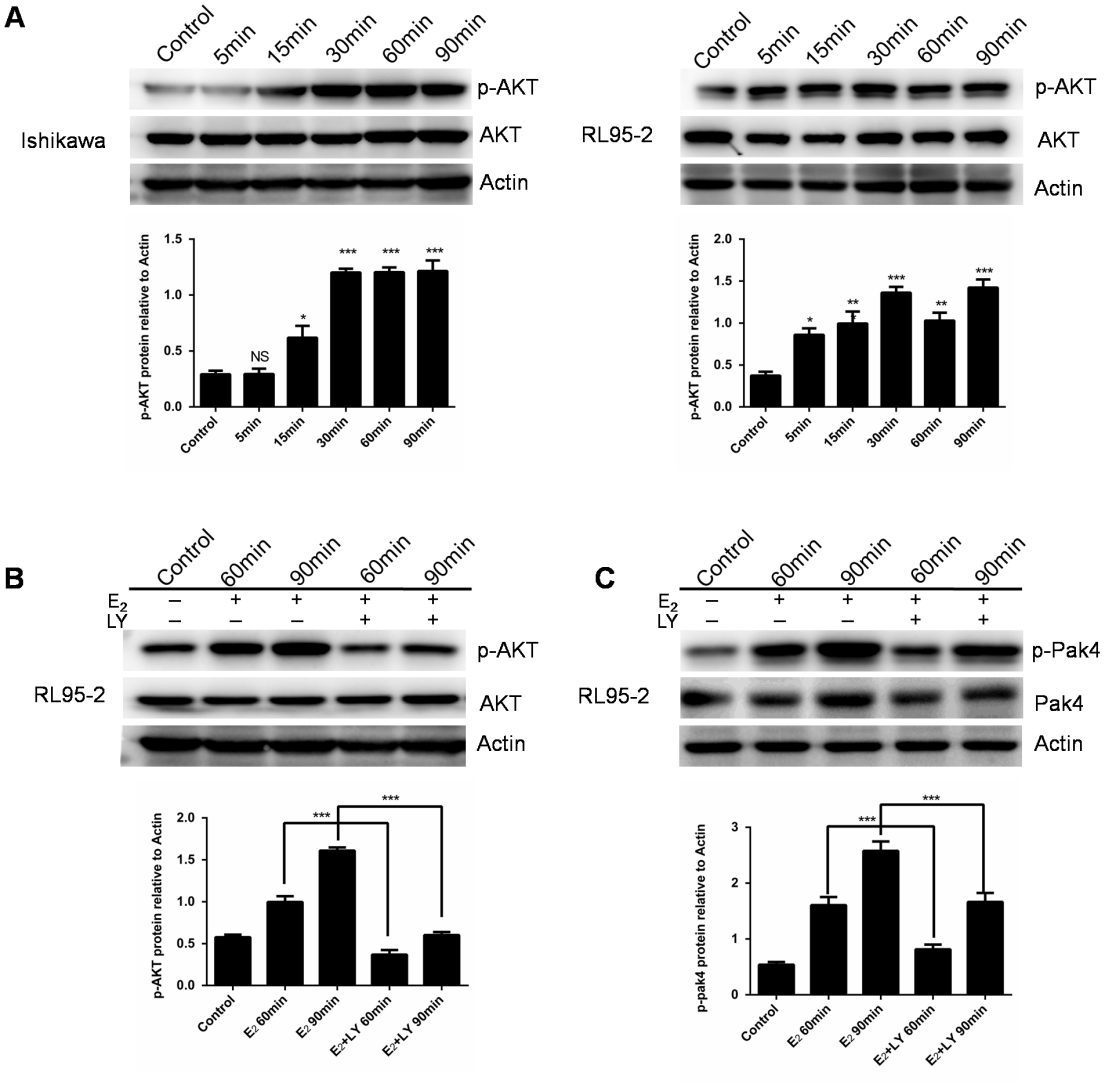
E_2_ activates Pak4 via PI3K/AKT pathway **(A)** Ishikawa and RL95-2 cells were treated with 10 nM E_2_ for up to 90 min, and Western blot was used to detect p-AKT Ser^473^ and total AKT levels. **(B-C)** RL95-2 cells were treated with E_2_ for 60 or 90 min in the presence or absence of 20 μM LY 294002. Upper panel **(B)** p-AKT Ser^473^ and **(C)** p-Pak4ser^474^ levels were determined by western blotting, using β-actin as a loading control. Lower panel: Densitometric analysis of **(B)** p-AKT and **(C)** p-Pak4 in the immunoblots. Values represent mean ± s.d. (n = 3). *P< 0.05, ***P<0.001 compared with control, according to t-test. All experiments were carried out in triplicates.

We next investigated the estrogen downstream signaling involved in the Pak4 activation. We found that estrogen increased AKT phosphorylation within 15min in Ishikawa cells, and 5 min in RL95-2 cells, and lasted for at least 90 min (Figure 2A). In order to elucidate the role of PI3K/AKT in estrogen-induced Pak4 activation, we treated RL95-2 cells with LY 294002, a specific PI3K inhibitor, in the presence of estrogen. LY 294002 significantly blocked the E_2_ mediated AKT stimulation, and partially blocked Pak4 phosphorylation (Figure 2B and 2C), suggesting that PI3K/AKT signaling mediates the estrogen-induced Pak4 activation.

### Estrogen induces Pak4 and p-Pak4 nuclear levels in EC cells

**Figure 3:**
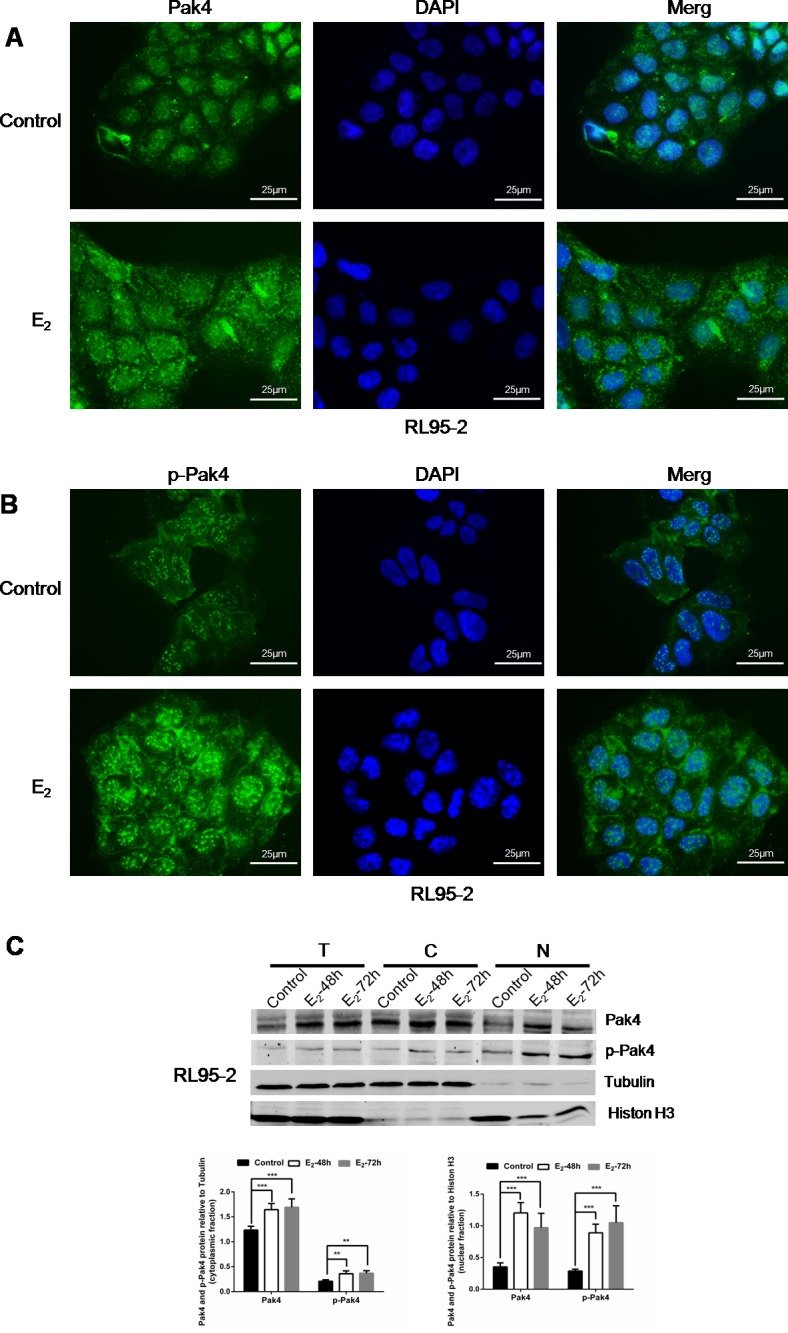
E_2_ promotes Pak4 and p-Pak4 nuclear accumulation Immunofluorescence staining of **(A)** Pak4 and **(B)** p-Pak4 in RL95-2 cells treated with or without E_2_. Original magnification×400, bar=25μm. **(C)** Immunoblot analyses of Pak4 and p-Pak4 in subcellular protein fractions extracted from RL95-2 cells (T, total celllysate; C, cytoplasmic fraction; N, nuclear fraction). Cells were serum-starved for 24 h, and then treated with 10 nM E_2_, for indicated times.

We have previously observed mild nuclear and strong cytoplasmic Pak4 levels, and strong nuclear and moderate cytoplasmic p-Pak4 levels in EC tissues [21]. In this study, we further investigated the subcellular localization of Pak4 and p-Pak4 in EC cells by immunofluorescence staining. As shown in Figure 3A and 3B, Pak4 was found in the cytoplasm and in the nucleus, whereas mild cytoplasmic and strong nuclear p-Pak4 immunostaining was observed in human RL95-2 endometrial cancer cells. Moreover, we observed that estrogen treatment stimulated Pak4 and p-Pak4 nuclear accumulation (Figure 3A and 3B). Increased expression of Pak4 and p-Pak4 in cytoplasmic and nuclear fractions of RL95-2 in the presence of E_2_ was also confirmed by western analysis (Figure 3C).

### Pak4 promotes ERα trans-activation

**Figure 4:**
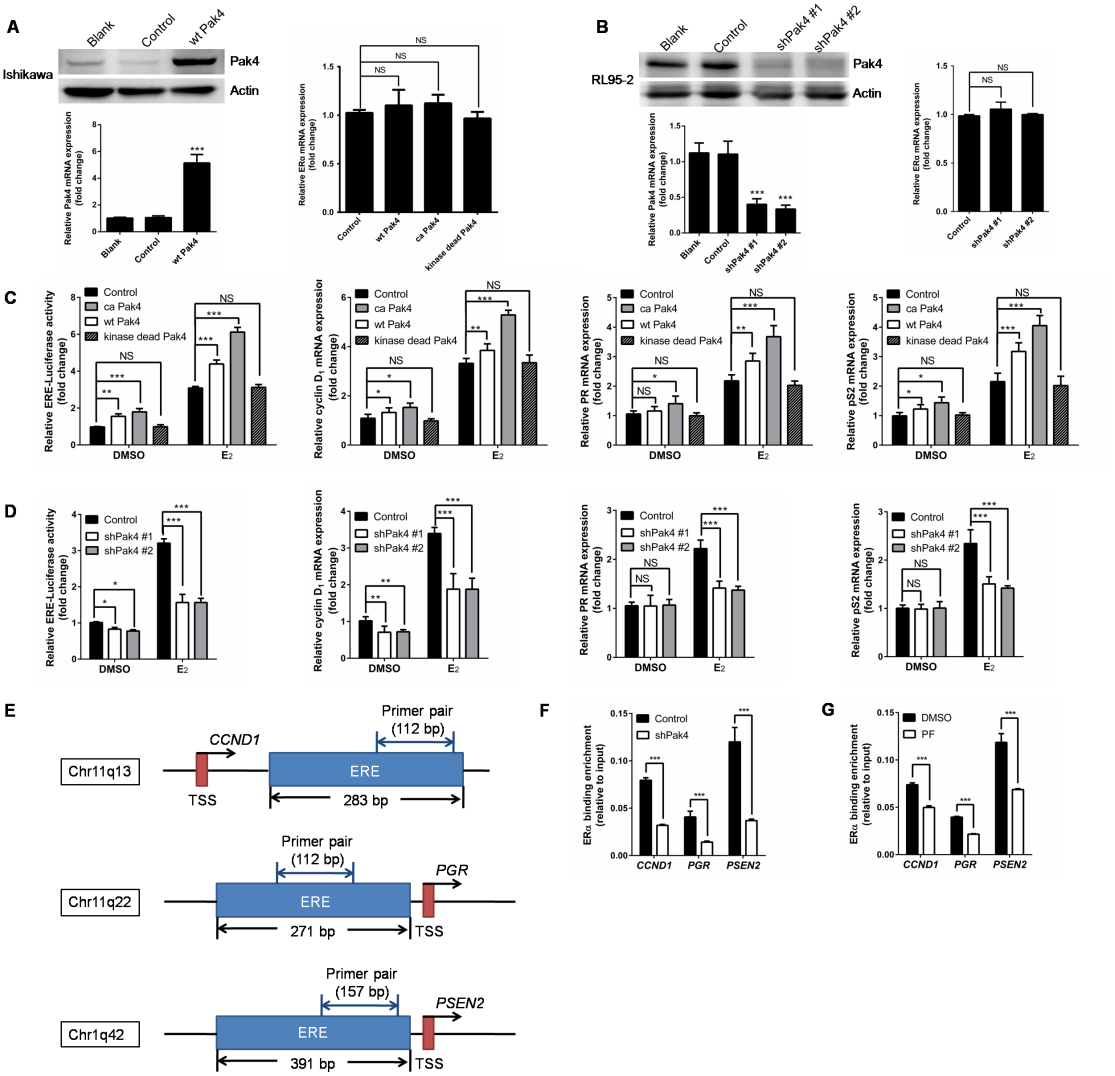
Pak4 enhances ERα transcription and ERα target gene expression **(A)** Left: Protein and mRNA levels of Pak4 were measured in wt Pak4-overexpressing Ishikawa cells by Western blot and qRT-PCR analysis, respectively; Right: ERα mRNA levels detected by qRT-PCR. Values are the mean ± SD from at least three independent experiments. **(B)** Left: Western blot and qRT-PCR of Pak4 levels in two different shPak4-transfected RL95-2 cells. Right: ERα mRNA levels detected by qRT-PCR. Values are the mean ± SD from at least three independent experiments. **(C)** Ishikawa cells were stably transfected with wt Pak4, ca Pak4, kinase-dead Pak4, or the control vector. **(D)** RL95-2 cells were stably transfected with two different shPak4 or the control vector. The ERE-Luc reporter plasmids were transfected into Ishikawa and RL95-2 cells 24 h before E_2_ treatment, and luciferase assay was performed 48 h after E_2_ addition. The mRNA levels of ERα target genes were determined by qRT-PCR. Cells were treated with 10 nM E_2_ or vehicle for 48 h before RNA extraction. Values represent mean ± s.d. (n = 3), from three independent experiments. *P< 0.05, **P<0.01, ***P<0.001 compared with control, according to t-test. **(E)** Schematic representation of the estrogen response element and the primers used for ChIP–qPCR. ERE: estrogen response element. TSS: transcription start sites. **(F-G)** A summary of ChIP-qPCR results for ERα binding in RL95-2 cells with the primer pairs shown in **(E)**. **(F)** shPak4 and control vector transfected RL95-2 cellswere treated with 10 nM E_2_ for 48 h before DNA extraction. **(G)** RL95-2 cells were treated with 10 nM E_2_ in the presence or absence of 1μM PF 3758309.

Following the nuclear accumulation of Pak4 and p-Pak4 after E_2_ stimulation, we then investigated the role of Pak4 in ERα trans-activation. Ishikawa cells that have relatively low Pak4 levels, were stably transfected with wild-type (wt) Pak4, constitutively active (ca) Pak4, or kinase-dead Pak4, whereas RL95-2 cells were stably transfected with two different shRNA constructs against human Pak4. The Pak4 mRNA and protein levels were substantially enhanced by wt Pak4 overexpression (Figure 4A) and reduced by Pak4 depletion (Figure 4B). Neither overexpression nor depletion of Pak4 affected the ERα mRNA levels (Figure 4A and 4B).

In order to elucidate the effect of abnormal Pak4 expression on ERα transcription, we performed an estrogen response element (ERE) luciferase assay. We found that wt Pak4 and ca Pak4 significantly increased ERE-dependent trans-activation of ERα in estrogen-treated Ishikawa cells (Figure 4C). Similarly, ca Pak4 also increased ERα target gene expression, including cyclin D1, PR, and pS2, both with and without E_2_ treatment. Wild-type Pak4 also induced mRNA expression of cyclin D1, PR, and pS2 in the presence of E_2_, but had no effect on PR expression in the absence of E_2_ (Figure 4C). Conversely, a stable knockdown of Pak4 decreased ERE luciferase activity in RL95-2 cells, both in the presence and absence of E_2_ (Figure 4D). Significantly reduced cyclin D1, PR, and pS2 mRNA levels were also observed in RL95-2 cells after shPak4 knockdown in the presence of E_2_. Interestingly, without E_2_ treatment, Pak4 inhibition only decreased cyclin D1 mRNA expression, but had no effect on PR and pS2 (Figure 4D).

To investigate whether Pak4 regulates the recruitment of ERα to ERE of target genes, we performed ChIP–qPCR using primers for ERE of *CCND1*, *PGR*, and *PSEN2* genes (Figure 4E). Consistent with the results above, knockdown of Pak4 with shRNA (Figure 4F) or inhibition of Pak4 with Pak4-inhibitor PF 3758309 (Figure 4G) impaired the recruitment of ERα to ERE of the target genes.

### Pak4 inhibition decreases E_2_-induced cell proliferation via alteration of G0/G1-phase cell cycle progression

**Figure 5:**
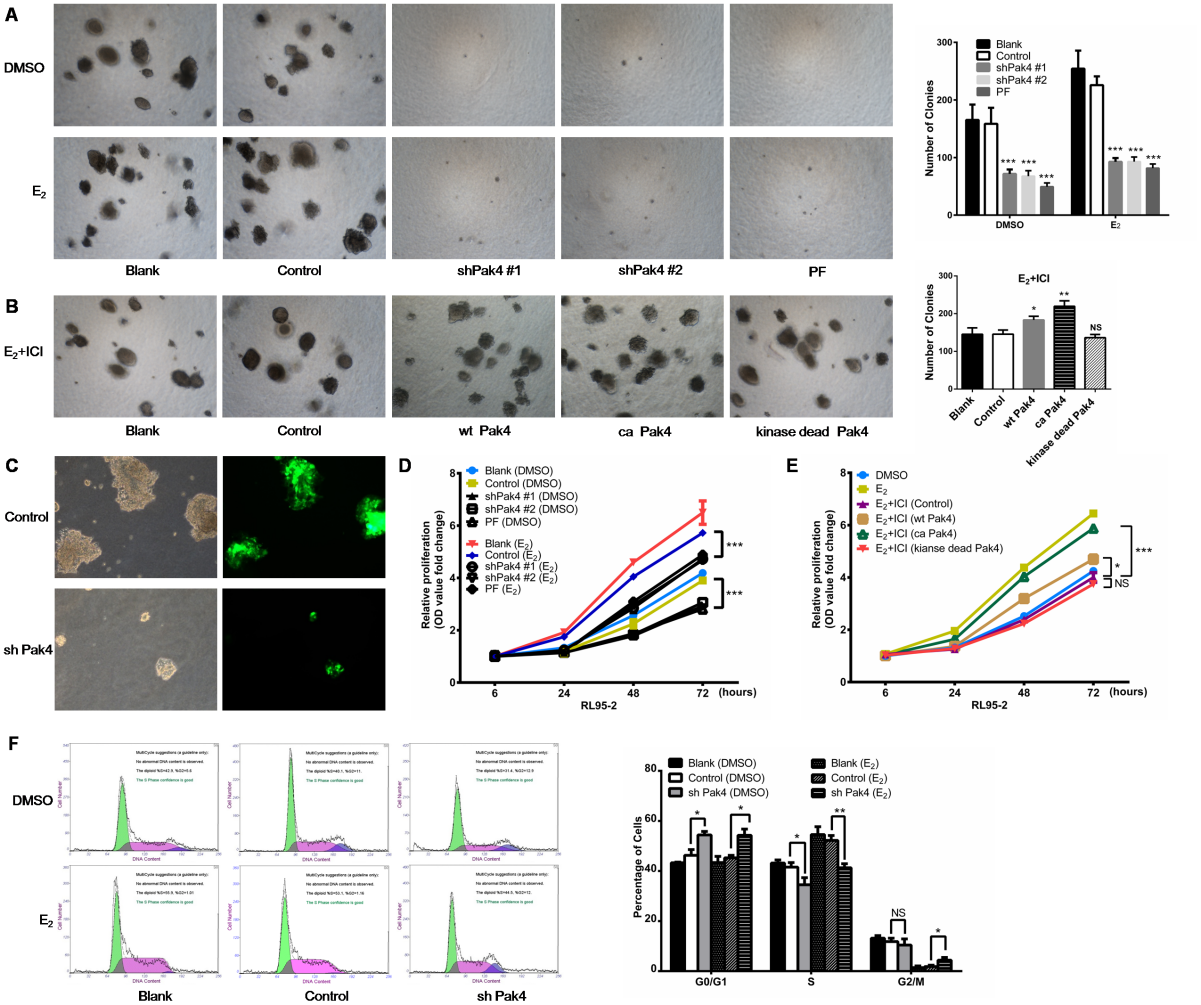
Pak4 inhibition suppresses E_2_-induced cell proliferation and cell cycle progression **(A)** Soft agar colony assays of Pak4 knockdown, Pak4 inhibitor PF 3758309 treated RL95-2 cells and control cells. Cells were cultured in the medium with or without E_2_ for 2 weeks. **(B)** RL95-2 cells were transfected with wt Pak4, ca Pak4, kinase-dead Pak4, or the control vector. Cells were cultured in the medium with 10 nM E_2_ and 100 nM ICI 182,780 for 2 weeks. Representative images (left) were captured with an inverted phase contrast microscope (magnification, ×200). Columns (right), represent the number of colonies from three independent experiments, each in triplicates; values represent mean ± s.d.; *P< 0.05, **P<0.01, ***P<0.001. **(C)** RL95-2 cells were stably transfected with shPak4 or control vector with GFP. The fluorescence images showing the transfection efficiency, as well as the decreased size of colonies in Pak4 knockdown cells compared with control cells. Original magnification, ×400. **(D)** MTT assay of Pak4 knockdown, Pak4 inhibitor PF 3758309 treated RL95-2 cells and control cells. Cells were either treated with E_2_, vehicle or left untreated as indicated. **(E)** RL95-2 cells were transfected with wt Pak4, ca Pak4, kinase-dead Pak4, or the control vector. Cells were treated with 10 nM E_2_, 10 nM E_2_+ 100 nM ICI 182,780, or vehicle as indicated. All experiments were carried out in triplicates. **(F)** Cell-cycle profiles of shPak4 RL95-2 cells were assessed by FACS using DNA content profiles (left). Cells were either treated with E_2_, vehicle, or left untreated for 96 h before measurement. The percentages of cells in each compartment were calculated (right). Values represent mean ± s.d. (n = 3). *P< 0.05, **P<0.01 compared with control, according to t-test. All experiments were carried out in triplicates.

We next investigated whether estrogen increases EC cell proliferation by activating Pak4. As shown in Figure 5A and 5C, E_2_ treatment promoted RL95-2 cell colony formation in soft agar, whereas depletion or functional inhibition of Pak4 (with Pak4 inhibitor PF 3758309) almost completely abrogated this effect. In addition, using MTT proliferation assay, we found that E_2_ induced RL95-2 cell proliferation, and that the E_2_-induced proliferation was reversed by shPak4 or PF 3758309 (Figure 5D). We next inhibited the estrogen pathway with a selective ERα inhibitor ICI 182,780, and then rescued the phenotype with overexpression of wt Pak4, ca Pak4, or kinase-dead Pak4. We observed that ICI 182,780 treatment inhibited the E_2_-induced colony formation, and overexpression of wt Pak4 or ca Pak4 partly rescued the number of colonies (Figure 5A and 5B). MTT proliferation assay provided similar results (Figure 5E).

To assess the mechanism of Pak4-induced cellular proliferation, the cell-cycle profiles of RL95-2 cells were determined using propidium iodide staining and fluorescence-activated cell sorter analysis. Knockdown of Pak4 in RL95-2 cells resulted in accumulation of cells in G0/G1 phase and a decrease in S phase compared with controls, both in the presence and absence of E_2_ (Figure 5F).

### Pak4 inhibition suppresses estrogen-induced tumor growth in nude mice

**Figure 6:**
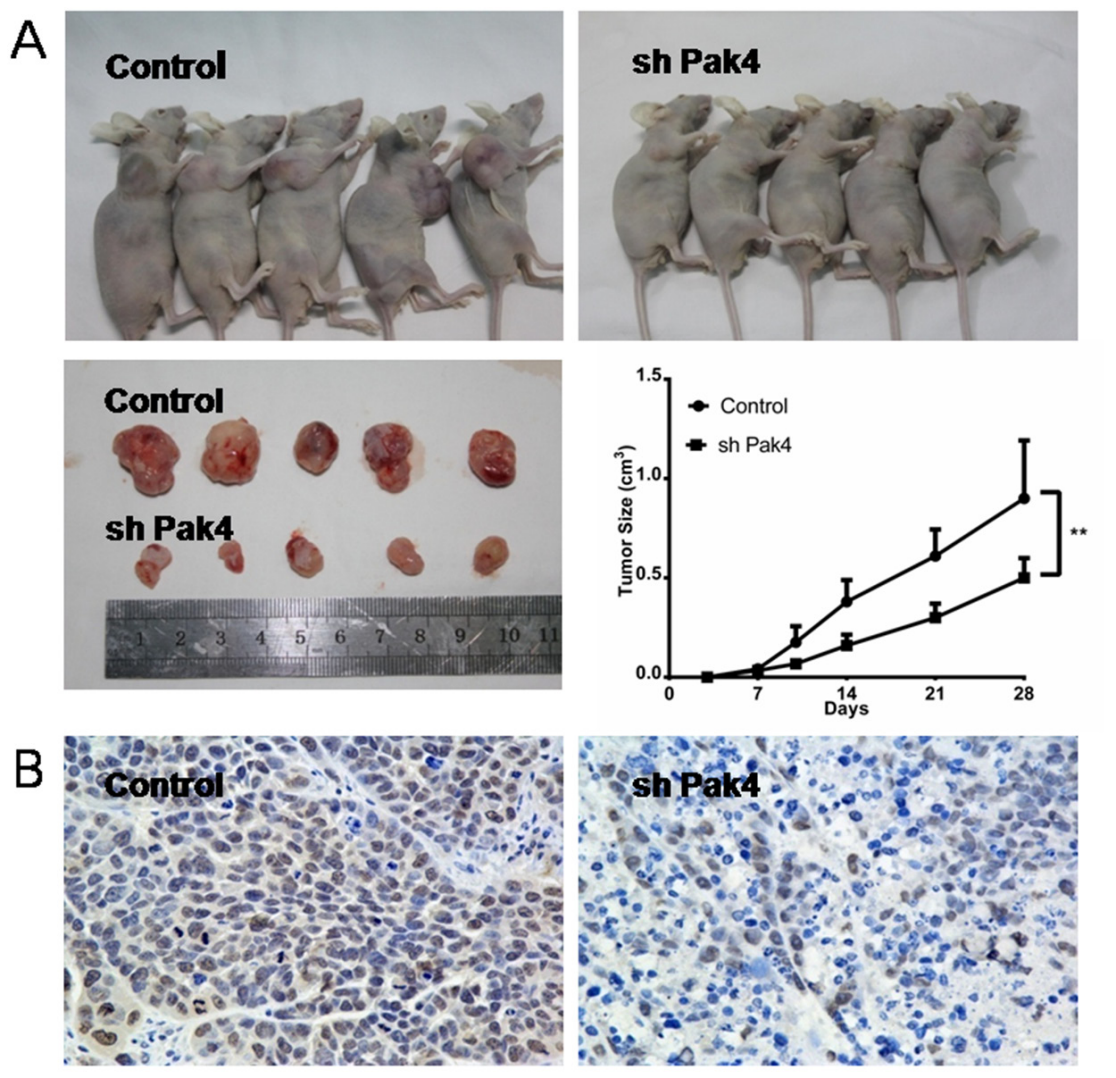
Pak4 depletion inhibits tumor growth *in vivo* **(A)** Growth rates of tumors in nude mice inoculated with shPak4 RL95-2 cells or control cells. Values represent mean ± s.d. (n = 5). **P<0.01 compared with control, according to t-test. **(B)** Immunohistochemical staining of Pak4 in control and shPak4 tumors.

To investigate the role of Pak4 *in vivo*, RL95-2 cells with stably knocked-down Pak4 (shPak4) or control cells were injected subcutaneously into the flanks of nude mice. Both groups were administered subcutaneously 17β-estradiol 90-day-release pellets. The growth rate of tumors in mice inoculated with shPak4 RL95-2 cells was significantly lower than in tumors formed by control cells (*P*=0.0198; Figure 6A). The Pak4 suppression in shPak4 tumors was confirmed by immunohistochemistry staining (Figure 6B).

## DISCUSSION

**Figure 7:**
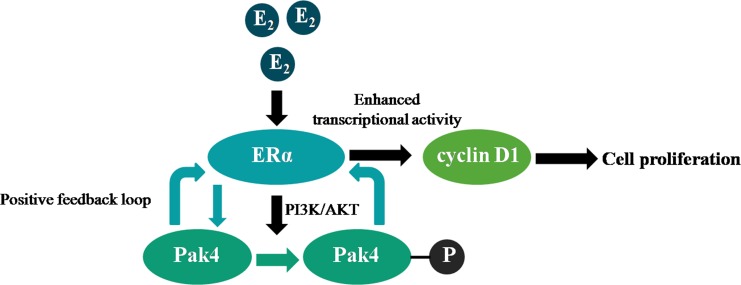
Illustration of a positive feedback loop between Pak4 and ERα signaling in endometrial cancer Estrogen increases Pak4 expression and activation via PI3K/AKT pathway, the increased and activated Pak4 in turn enhances ERα transcriptional activity and cyclin D1 expression, which facilitates EC cell proliferation.

Cross-talk between ERα and signaling pathways contributes to the pathogenesis of endometrial carcinoma [22], but the underlying mechanisms are not clear. In the present study, we identified a positive feedback loop between Pak4 kinase and ERα signaling that promotes EC cell proliferation, and determined the role of PI3K/AKT signaling pathway and cyclin D1 in the process (Figure 7).

Pak4 is overexpressed and/or activated in various human cancer cells [16, 23, 24]. It is upregulated in cancers of breast, stomach, ovary, pancreas, and prostate [12, 25–30]. Although in our previous study, we observed overexpression and activation of Pak4 in estrogen-induced EC cells, it was not clear whether it was related to estrogen signaling [21]. In this study, we found that the expression and activation of Pak4 was increased in response to estrogen stimulation in EC cells, and that the activation occurred via PI3K/AKT pathway. In HGF signaling, Pak4 is activated through PI3K in MDCK cells [17]. However, LY294002, the inhibitor of PI3K, had no effect on HGF-induced Pak4 activation in prostate cancer cell lines [29]. In this study, we observed that LY294002 treatment partially blocked E_2_-induced Pak4 phosphorylation. These findings suggest that the relationship between Pak4 and PI3K/AKT pathway may be cell specific.

In the present study, E_2_ treatment also induced Pak4 phosphorylation, suggesting that in addition to the increased expression, the activation of Pak4 may play an important role in EC progression. In our previous study, we found a positive correlation between nuclear p-Pak4 expression and EC progression [21]. Here, we observed Pak4 and p-Pak4 nuclear accumulation after E_2_ stimulation. We also demonstrated that wild-type Pak4 increased ERα trans-activation in the presence or absence of estrogen. A more pronounced effect was observed when cells were transfected with constitutively active Pak4, suggesting that Pak4 may activate ERα in an estrogen-independent manner, and may contribute to the development of hormone independence. Therefore, the protein level and activation of Pak4 may potentially affect the effectiveness of anti-estrogen therapies; this is consistent with previous reports of Pak1 signaling-dependent activation of ER-S305 leading to enhanced S118 phosphorylation, and development of tamoxifen resistance in breast cancer [11]. Another study reported that GNE-2861, a small molecule selectively inhibiting group II PAKs, overcomes tamoxifen resistance in breast cancer [12]. Therefore, the emergence of Pak4 inhibitors [31, 32] suggests that targeting Pak4 expression or activity may represent a novel strategy to increase the response to hormonal treatment in endometrial cancer.

Furthermore, we demonstrated that depletion or functional inhibition of Pak4 abrogated cell proliferation induced by E_2_, whereas increased Pak4 expression and activation rescued the impaired cell proliferation caused by inhibiting the estrogen pathway. We also demonstrated that Pak4 knockdown decreased cyclin D1 expression with and without E_2_ treatment. Cyclin D1, a D-type cyclin regulating G1-phase cell-cycle progression, has been identified as a critical downstream effector of estrogen signaling and is frequently overexpressed in endometrial cancer [33–36]. Pak4 has been shown to control cell cycle progression by regulating the cyclin-dependent kinase inhibitor p21 [37]. Downregulation of Pak4 decreases proliferation and increases apoptosis and S phase arrest in Hep-2 cells via activation of the ATM/Chk1/2/p53 pathway [38]. Our results show that knockdown of Pak4 induces G1-S arrest, which is consistent with the effect of cyclin D1 downregulation [39].

In summary, our results demonstrate the presence of a positive feedback loop between Pak4 and ERα signaling in endometrial cancer. Estrogen stimulation leads to increased Pak4 expression, as well as hyper-activation via PI3K/AKT pathway, and the increased and activated Pak4 in turn enhances ERα trans-activation. This positive feedback loop promotes endometrial cancer cell proliferation by increasing cyclin D1 expression and altering cell cycle progression. The correlation between Pak4 and ERα signaling not only reveals an underlying mechanism of estrogen-related tumor progression, but also provides a rationale for multi-targeted therapies against endometrial cancer.

## MATERIALS AND METHODS

### Cell culture

Ishikawa, RL95-2, and MCF-7 cells were purchased from American Type Culture Collection (Manassas, VA). Cells were maintained in DMEM/F12 (Gibco, Auckland, NZ) supplemented with 10% FBS (Gibco, Carlsbad, CA) in a 37°C, 5% CO2 incubator.

### Plasmids and transfection

The Pak4 mutants including ca Pak4 (E474) and kinase-dead Pak4 (M350) were generated by site-specific mutagenesis from the Pak4 wild-type using the Quick Change Site Directed Mutagenesis Kit (Stratagene, LaJolla, CA, USA). The ca Pak4 mutant was generated by mutating Ser474 to glutamic acid. The resulting Pak4 (E474) mutant showed enhanced auto-phosphorylation activity [40]. The kinase-dead Pak4 (M350) mutant carries a mutation in which the conserved lysine in subdomain II is converted to a non-phosphorylated methionine, resulting in a completely inactive kinase [40].

To stably express Pak4 in Ishikawa cells, wt Pak4, ca Pak4, kinase-dead Pak4, or the control vector pEGFP-N1 (Clontech Laboratories, Palo Alto, CA) were transfected into cells at 70% confluency in 12-well culture plates, using Lipofectamine 2000 (Invitrogen, Carlsbad, CA) and then selected with G418 (800 μg/mL, SIGMA Chemical, St Louis, MO, USA). To stably silence Pak4 in RL95-2 cells, cells were transfected with two different shRNA constructs against human Pak4 or the control vector psiHIV-U6 (GeneCopoeia, Germantown, MD), and then selected with puromycin (0.5μg/mL, SIGMA Chemical, St Louis, MO, USA).

### Estrogen treatment, PI3K, Pak4 and ERα inhibitor treatment

For western blot analysis, cells were seeded in 6-well plates at 70% confluency and cultured for 24 h in serum and phenolred-free medium, then treated with 10 nM 17β-estradiol (SIGMA Chemical, St Louis, MO, USA) or vehicle (DMSO, 0.1%) for the indicated times. For long-term treatment, cell were seeded in 6-well plates, at 70% confluency at different time points, cultured in a medium containing 10% charcoal-stripped FBS (HyClone, Logan, UT). Cells were plated for 24 h before treatment with the PI3K inhibitor LY 294002 (20 μM, SIGMA Chemical, St Louis, MO, USA), Pak4 inhibitor PF3758309 (1μM, Selleck, Shanghai, CHINA), ERα inhibitor ICI 182,780, (100 nM, SIGMA Chemical, St Louis, MO, USA), with DMSO as control.

### Biochemical fractionation

Cytoplasmic and nuclear extracts from RL95-2 cells were isolated using the NE-PER Nuclear and Cytoplasmic Extraction Reagents (Pierce; Thermo Scientific, Rockford, IL, USA). Primary antibodies were the following: rabbit anti-Pak4 (1:1000, Abcam, Cambridge, UK), rabbit anti-p-Pak4 Ser^474^ (1:1000, Cell Signaling Technology), mouse anti-β-actin (1:2000, ProteinTech Group, Chicago, IL), rabbit anti-tubulin (1:1000, Cell Signaling Technology), and rabbit anti-histone H3 (1:1000, Cell Signaling Technology).

### Luciferase reporter assays

The reporter gene ERE-Luc was constructed using the enhanced luciferase reporter gene pGL3-promotor. Three tandem repeats of the consensus ERE oligo (GGTCACTGTGACC) were inserted into the Mlu I-Bgl II site of the multiple cloning site of pGL3-promotor, upstream of SV40 promoter. The ERE-Luc reporter and phRL/CMV (Renilla luciferase) plasmids were co-transfected into Pak4 overexpressing Ishikawa cells and Pak4 knockdown RL95-2 cells. After 24 h, cells were treated with or without E_2_. Luciferase assay was performed using Dual-Luciferase Reporter Assay System (E1531, Promega, Mannheim, Germany) after 48 h treatment with E_2_.

### Chromatin immunoprecipitation (ChIP)–PCR

The ChIP assay was performed in RL95-2 cells using the Pierce Agarose ChIP kit (Pierce; Thermo Scientific, Rockford, IL, USA) following the manufacturer's instructions. The antibody used in ChIP analysis was anti-ERα (1:50, Epigentek Group Inc, Brooklyn, NY, USA). Specific primers for qRT-PCR were: *CCND1*: 5’-CGCTTCCCAGCACCAACA-3’, 5’-CAAAGAGGCAGGCACCAC-3’; *PSEN2*: 5’-ATGT GAGAACAACCGGGAGGA-3’, 5’-TCGGAACTAAGC GACGACCTT-3’; *PGR*: 5’-GAGAAAGTGGGTGTTG AATGTG-3’, 5’-TGACGACAGGATGGAGGC-3’. Enrichment was calculated using the comparative Ctmethod. IgG was used as a negative control.

### Soft agar colony assay

Cells were seeded on 0.3% top agar in a growth medium over a layer of 0.6% agar in a 6-well plate, at a density of 1×10^4^ cells/mL (100 μL/well). Growth medium with or without E_2_ or E_2_+ICI 182,780 or PF 3758309 was added to the wells every 3 to 4 days. After 2 weeks of incubation, colonies of more than 50 cells were produced. Colonies were photographed and counted with an inverted microscope.

### RNA extraction and qRT-PCR

Total RNA was extracted from cell lines using TRIzol reagent (Invitrogen, Life Technologies; Shanghai, PR China). cDNA was reverse-transcribed from total RNA using Prime Script RT reagent Kit (Takara, Dalian, PR China). Real-time PCR was performed using SYBR Premix Ex Taq (Takara, Dalian, PR China) and analyzed with an ABI Prism 7000 Sequence Detection System. The oligonucleotide primers used were: Pak4: 5’-ATGTGGTGGAGATGTACAACAGCTA-3’, 5’-GTTCATCC TGGTGTGGGTGAC-3’. The primers for ERα, cyclin D1, PR, and pS2 were described previously [41]. Gel electrophoresis was used to confirm PCR purity. All data were obtained in triplicates in three independent experiments.

### Western blot

Cells were lysed using ProteoJET Mammalian Cell Lysis Reagent (MBI Fermentas, ON, Canada) with a protease inhibitor cocktail (Roche Diagnostics, Basel, Switzerland). Total protein concentration was estimated using the BCA method (Pierce, Rockford, IL, USA). A total of 60 μg of protein was separated on an 8% sodium dodecyl sulfate–polyacrylamide gel and transferred to a polyvinylidene fluoride (PVDF) membrane. Membranes were incubated with primary antibodies. Signal was detected using the BeyoECL Plus (Beyotime, Shanghai, PR China).

Primary antibodies were: rabbit anti-Pak4 (1:1000, Abcam, Cambridge, UK), rabbit anti-p-Pak4 Ser^474^ (1:1000, Cell Signaling Technology), mouse anti-β-actin (1:2000, ProteinTech Group, Chicago, IL), rabbit anti-AKT1 (1:1000, Epitomics, Burlingame, CA, USA), and rabbit anti-p-AKT Ser^473^ (1:1000, Epitomics, Burlingame, CA, USA).

### Cell cycle analysis

Cells were fixed in 70% ice-cold ethanol. The cells were stained with 25μg/mL propidium iodide (KeyGen Biotech, Shanghai, PR China) in fluorescence-activated cell sorting buffer (PBS containing 0.1% bovine serum albumin, 0.05% of Triton X-100, and 50 μg/mL of RNaseA). After incubation for 30 min at room temperature, the cells were analyzed by flow cytometry (Becton Dickinson FACScan). Tests were performed in triplicates.

### Proliferation assay

Cells (2 ×10^3^cells/well) were seeded in 96-well culture plates in a growth medium. Cell viability was measured every 24 h by MTT assay following the manufacturer's instructions (Beyotime, Shanghai, PR China). Medium was changed every other day. Each experiment was repeated in triplicates.

### Cell immunofluorescence

Cells treated with or without E_2_ were seeded on coverslips and cultured in DMEM/10% FBS overnight. Cells were fixed with 4% paraformaldehyde, permeabilized with 0.1% Triton X-100 and blocked with goat serum before incubation with antibodies against Pak4 (1:100, Abcam), or p-Pak4 Ser^474^ (1:100, Cell Signaling Technology) at 4°C overnight. The cells were incubated with Alexa 594-labeled secondary antibodies (1:200; Invitrogen, Burlington, ON, Canada), and counterstained with 2-(4-Amidinophenyl)-6-indolecarbamidine dihydrochloride (DAPI; Beyotime) before analysis using a microscope (Leica TCS SP8). The control slides were treated with PBS instead of the primary antibody.

### Studies *in vivo*

The 5 × 10^6^ shPak4 or vector-transfected RL95-2 cells were inoculated s.c. (five mice per group) into four-week-old BALB/c female nude mice. Estrogen was administered to the animals subcutaneously as 17-beta-estradiol 90-day-release pellets (0.72 mg/pellet; IRA, Toledo, OH) as described previously [42]. The diameters of s.c. tumors were measured perpendicularly weekly, and volumes were calculated using the following standard formula: tumor volumes (cm^3^) = (the longest diameter) × (the shortest diameter)^2^× 0.5. Mice were sacrificed at 28 days post-injection. Tumors were excised and measured. All experimental protocols were approved by the Ethics Committee for Animal Experimentation at Tongji University.

### Statistical analysis

All statistical analyses were performed using SPSS 16.0 (Microsoft, Redmond, WA, USA) or Prism (GraphPad, San Diego, CA, USA). Each experiment was performed as least three times, and data were expressed as the means ± SD where applicable. A *P* value < 0.05 was considered to be significant.
